# The Mechanisms Underlying the Cytotoxic Effects of Copper Via Differentiated Embryonic Chondrocyte Gene 1

**DOI:** 10.3390/ijms20205225

**Published:** 2019-10-22

**Authors:** Ssu-Yu Chen, Shu-Ting Liu, Wun-Rong Lin, Chi-Kang Lin, Shih-Ming Huang

**Affiliations:** 1 Department of Biochemistry, National Defense Medical Center, Taipei 114, Taiwan; windblowvoice@gmail.com (S.-Y.C.); shuting0719@gmail.com (S.-T.L.); 2 Department of Urology, Mackay Memorial Hospital, Taipei 104, Taiwan; vincent751051@gmail.com; 3 Department of Medicine, Mackay Medical College, New Taipei 252, Taiwan; 4 Department of Cosmetic Applications and Management, Mackay Junior College of Medicine, Nursing, and Management, Taipei 112, Taiwan; 5 Department of Obstetrics and Gynecology, Tri-Service General Hospital, National Defense Medical Center, Taipei 114, Taiwan

**Keywords:** copper sulfate, cytotoxicity, reactive oxygen species, disulfiram, differentiated embryonic chondrocyte gene 1

## Abstract

Copper is an essential trace element within cells, but it also exerts cytotoxic effects through induction of reactive oxygen species (ROS) production. To determine the mechanisms underlying copper-induced ROS production, we examined the effects of copper sulfate in HeLa cells. Exposure to copper sulfate led to dose-dependent decreases in HeLa cell viability, along with increases in the subG1 and G2/M populations and corresponding decreases in the G1 population. Copper sulfate also increased the levels of apoptosis, senescence, mitochondrial dysfunction, autophagy, ROS, and the expression of several stress proteins, including ATF3, c-Fos, DEC1 (differentiated embryonic chondrocyte gene 1), p21, p53, and HIF-1α (hypoxia-inducible factor 1 alpha). The suppression of copper-induced ROS generation by the ROS scavenger *N*-acetyl cysteine verified copper’s functional role, while the suppression of copper’s effects by the copper chelator disulfiram, confirmed its specificity. Selective induction of HIF-1α, p53, and phosphorylated ERK proteins by copper was blocked by the knockdown of the transcription factor DEC1, suggesting copper’s effects are mediated by DEC1. In addition to HeLa cells, copper also exerted cytotoxic effects in human endometrial (HEC-1-A) and lung (A549) adenocarcinoma cells, but not in normal human kidney (HEK293) or bronchial (Beas-2B) epithelial cells. These findings shed new light on the functional roles of copper within cells.

## 1. Introduction

Copper (Cu) is an essential trace element that plays a central role in the biochemistry of every living organism [1,2,3,4,5]. Within organisms, copper is present in both oxidized (copper (II)) and reduced (copper (I)) forms, enabling it to serve as a co-factor in redox reactions that have been catalyzed by enzymes, such as cytochrome c oxidase, lysyl oxidase, and superoxide dismutase (SOD). The high redox reactivity of copper can also be a source of reactive oxygen species (ROS), which makes copper potentially cytotoxic. However, intracellular metallochaperones have evolved to collect copper from the transporters, mediating its cellular uptake and distributing it to target sites within cells via direct interaction with their target proteins.

The elevated copper concentrations correlate with cancer stage and/or progression in diverse types of tumors. This suggests that copper may be a useful prognostic factor and/or marker of responsiveness to therapy [6,7,8,9]. In addition, elevated copper (250–500 μM) induces premature senescence in some cell types (e.g., human diploid fibroblasts and fetal lung fibroblasts), which can be attenuated by the copper chelator resveratrol [10,11,12,13]. Copper also plays a central role in angiogenesis via the hypoxia-inducible factor 1α (HIF-1α)-vascular endothelial growth factor (VEGF) pathway [9,14,15]. Consequently, controlling copper levels with the aid of a copper chelator may have anti-angiogenic activity.

Appropriate intracellular ROS levels are normally maintained with the aid of scavenging systems, such as catalase, SODs, and other antioxidant defense systems [16,17,18,19]. However, the balance between the ROS and antioxidant species is disturbed in cancers. The major cellular ROS source is the mitochondrial respiration pathway, but other sources of ROS production are in the cytosol, where ROS formation is a consequence of aerobic metabolism. ROS appear to be involved in each step of cancer development, including its initiation, promotion, and progression. Elevated oxidative stress within cells can lead to the modification of a number of cellular targets, including nuclear and mitochondrial DNA, the mitochondrial inner membrane, and membrane phospholipids. Therefore, a number of studies have examined the possibility of exploiting elevated oxidative stress as a potential cancer treatment strategy. Such a strategy would entail inhibiting antioxidant enzymes or increasing ROS production within cancer cells [6,7,8,9,20].

Normal cells exhibit only low levels of ROS and have the reserve capacity to cope with oxidative insults. Whereas, cancer cells under conditions of sustained oxidative stress generally tend to be heavily dependent on adaptation mechanisms, and may exhaust ROS-buffering capacity [20]. In the present study, we examined the effects of copper sulfate exposure on human cervical adenocarcinoma (HeLa) cells, focusing on their ability to induce ROS generation. Our findings provide novel insight into the functional roles of copper-containing compounds within cells, as well as their potential antitumor applications.

## 2. Results

### 2.1. The Cytotoxic Effects of Copper Sulfate in HeLa Cells

**Figure 1:**
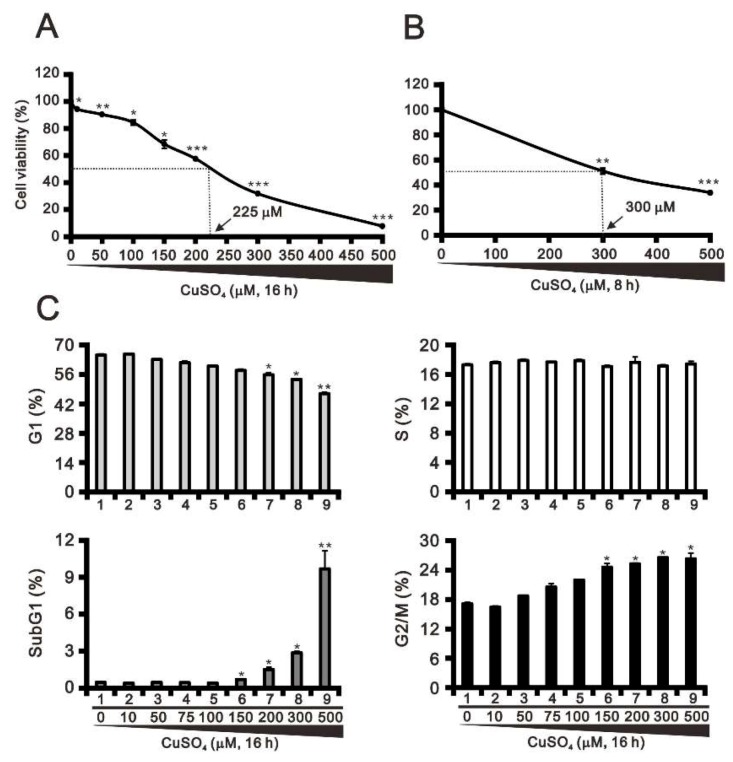
Effects of copper sulfate on cell viability and the cell cycle profile. (**A**,**B**) HeLa cells were treated with the indicated concentrations of copper sulfate for 16 h (**A**) or 8 h (**B**). Cell viability was measured using the MTT method. (**C**) HeLa cells were incubated with the indicated concentrations of copper sulfate for 16 h and then subjected to flow cytometric cell cycle profile analysis. The results are representative of three independent experiments. * *p* < 0.05, ** *p* < 0.01, and *** *p* < 0.001.

The cytotoxicity of copper has been observed under both physiological and pathological conditions [2,21]. Here, we examined the relative cytotoxicity of copper sulfate in HeLa cells at two treatment times. Our results indicate that the IC50 for copper sulfate is 225 μM and 300 μM after 16 h and 8 h of exposure, respectively (Figure 1A,B). We next monitored the cells treated for 16 h to assess the dose-dependent effects of copper sulfate on the cell cycle profile. We found that increasing the concentration of copper sulfate led to successive significant increases in the subG1 and G2/M populations, and decreases in the G1 population (Figure 1C, compare at 300 μM). This suggests that the cytotoxicity of copper sulfate may lead to an apoptotic response in HeLa cells.

**Figure 2:**
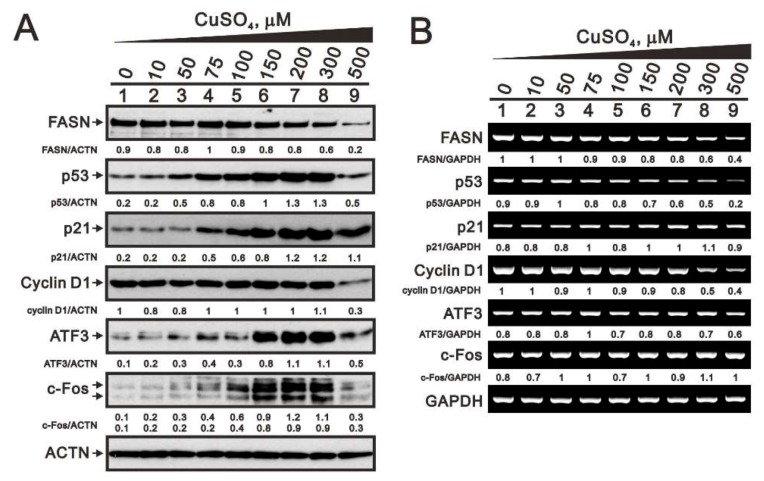
Responsiveness of copper-related factors. (**A**,**B**) HeLa cells were incubated for 16 h with the indicated concentrations of copper sulfate. (**A**) Western blot analysis of FAS, p53, p21, cyclin D1, ATF3, and c-Fos protein expression. (**B**) RT-PCR analysis for *FAS*, *p53*, *p21*, *cyclin D1*, *ATF3*, and *c-Fos* mRNA expression. ACTN was the protein loading control; *GAPDH* mRNA was the mRNA loading control. The results are representative of three independent experiments. Protein and PCR bands were quantified through pixel density scanning and evaluated using ImageJ, version 1.44a (http://imagej.nih.gov/ij/). The fold (shown above the bands) was normalized to the internal control protein (ACTN) or gene (*GAPDH*).

Previous studies have shown that copper induces cellular stress proteins, such as p53, ATF3, and c-Fos [15,22,23]. Accordingly, we assessed the expression levels of p53, p21, cyclin D1, ATF3, and c-Fos protein and mRNA in HeLa cells, that have been exposed to various concentrations of copper sulfate (Figure 2). Fatty acid synthase (FASN) protein and mRNA, which are well-known to be downregulated targets of copper, served as positive internal controls. HeLa cells were apparently dead at the highest concentration (500 μM) of copper sulfate, which is consistent with the subG1 population in Figure 1C. The levels of p53, p21, ATF3, and c-Fos protein were all increased in a dose-dependent manner by copper, while the levels of FASN tended to decrease (Figure 2A). By contrast, there was no consistent corresponding effects on mRNA levels. Whereas, the levels of *FASN*, *p53*, and *cyclin D1* mRNA tended to decline at higher concentrations of copper sulfate, while the levels of *p21*, *ATF3*, and *c-Fos* mRNA were unchanged (Figure 2B).

### 2.2. Effects of Copper Sulfate on ROS, Senescence, and Mitochondrial Membrane Potential in HeLa Cells

**Figure 3:**
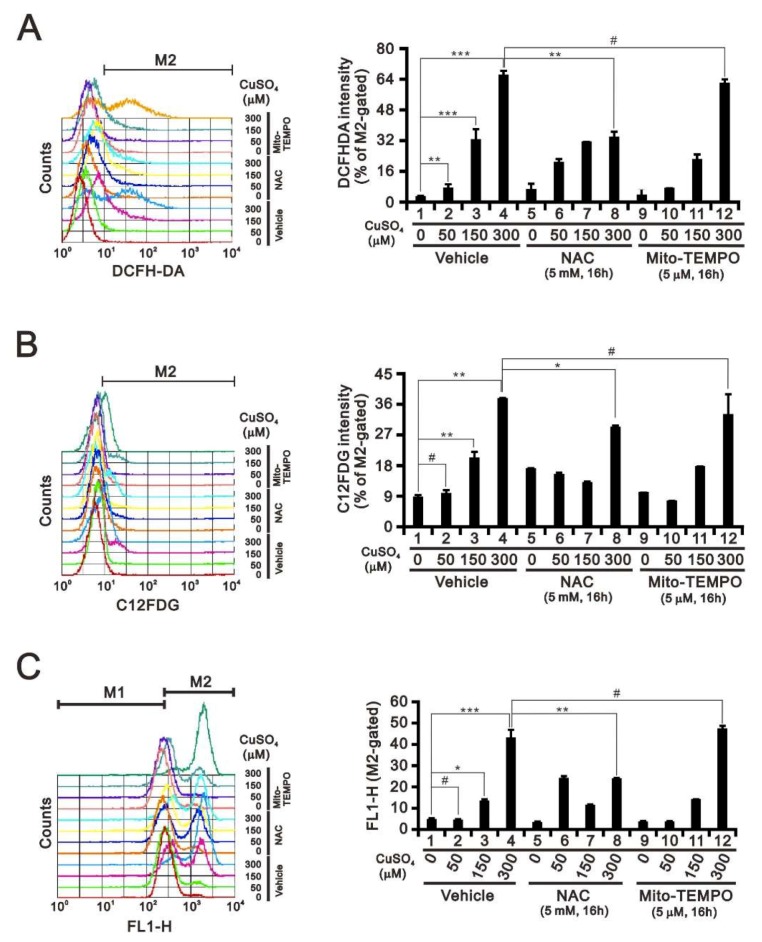
Effects of copper sulfate on the ROS, senescence, and mitochondrial membrane potential. (**A**–**C**) HeLa cells were incubated with the indicated concentrations of copper sulfate for 16 h in the presence of 5 mM NAC or 5 μM Mito-TEMPO, after which the live cells was stained with (**A**) 10 μM DCFH-DA; (**B**) 33 μM C12FDG; or (**C**) JC-1 and assayed using a flow cytometer. The results are representative of three independent experiments. # *p* > 0.05, * *p* < 0.05, ** *p* < 0.01, and *** *p* < 0.001.

ROS are generally divided into two subgroups: Free radicals, such as superoxide, and non-radicals, such as hydrogen peroxide (H_2_O_2_) [17,18]. Fenton-type reactions are important metal-mediated reactions, by which oxidation of a metal (usually a transition metal such as iron (II) or copper (I)) by H_2_O_2_ leads to the generation of a hydroxyl radical. As the most important source of cellular ROS production are mitochondria, we examined the effects of copper on ROS, cellular senescence, and mitochondrial membrane potential using flow cytometry with DCFHDA, C12FDG, and JC-1, respectively (Figure 3). Our data show that exposing HeLa cells to copper sulfate led to dose-dependent increases in ROS levels (Figure 3A) and cellular senescence (Figure 3B), as well as the disruption of the mitochondrial membrane potential (Figure 3C). Using 300 μM copper sulfate, we observed that the cytosolic ROS scavenger NAC (*N*-acetyl cysteine) partially inhibited those effects. Whereas, the mitochondrial ROS scavenger Mito-Tempo failed to affect them. These results suggest the primary effect of copper is to increase cytosolic ROS levels.

### 2.3. Effects of Copper Sulfate on Mitochondrial Bioenergetics in HeLa Cells

**Figure 4:**
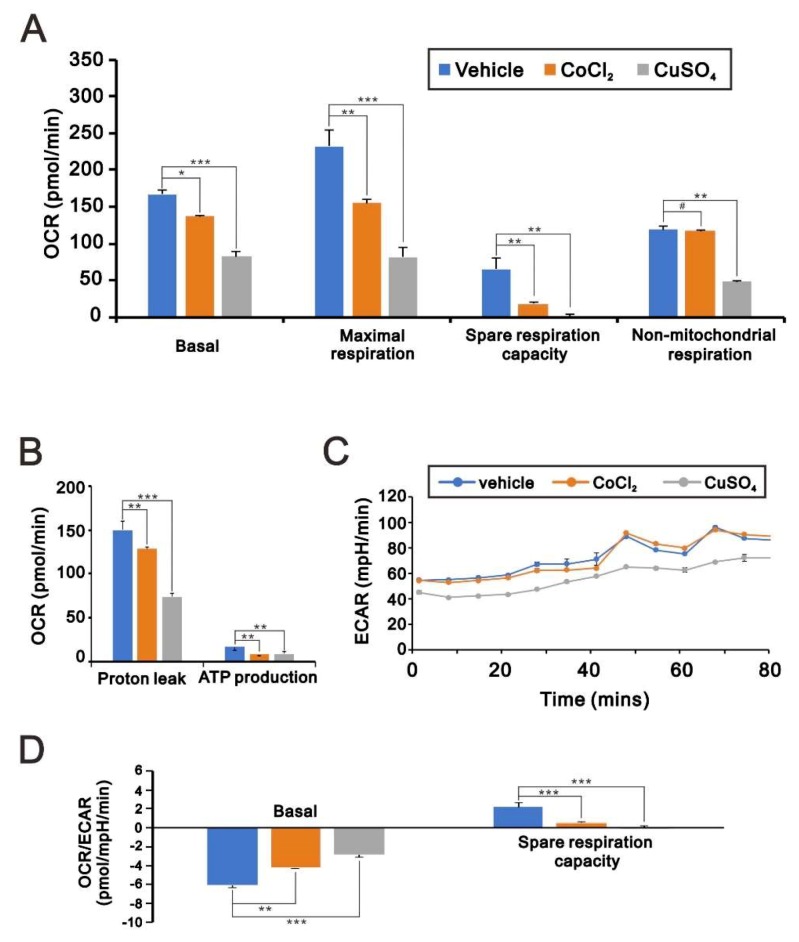
Effects of copper sulfate on oxygen consumption rate (OCR) and extracellular acidification rate (ECAR). (**A**–**D**) HeLa cells were incubated for 16 h with 200 μM CoCl_2_ or 100 μM CuSO_4_, after which the cellular OCR (**A**,**B**) and ECAR (**C**,**D**) were measured in Seahorse XF24 bioenergetic assays. # *p* > 0.05, * *p* < 0.05, ** *p* < 0.01, and *** *p* < 0.001.

The disruption of mitochondrial membrane potential by copper suggests that copper may also disrupt mitochondrial respiration and glycolysis in HeLa cells. We measured the changes in the oxygen consumption rate (OCR) and the extracellular acidification rate (ECAR) using an Agilent *Seahorse* XF Cell Mito Stress Test Kit (Figure 4). Previous studies showed that cobalt chloride induces mitochondrial dysfunction [24]. We found that cobalt chloride reduced mitochondrial respiration and the OCR (Figure 4A,B), as well as the ECAR (Figure 4C) and the OCR/ECAR ratio (Figure 4D) in HeLa cells. Notably, these effects were even more pronounced in the presence of copper sulfate. In addition, copper also suppressed non-mitochondrial respiration in HeLa cells. Thus, it appears that, to a significant extent, the cytotoxicity of copper sulfate can be attributed to mitochondrial dysfunction.

### 2.4. The Effects of Copper-Containing Compounds on the Stability of HIF-1α Protein in HeLa Cells

**Figure 5:**
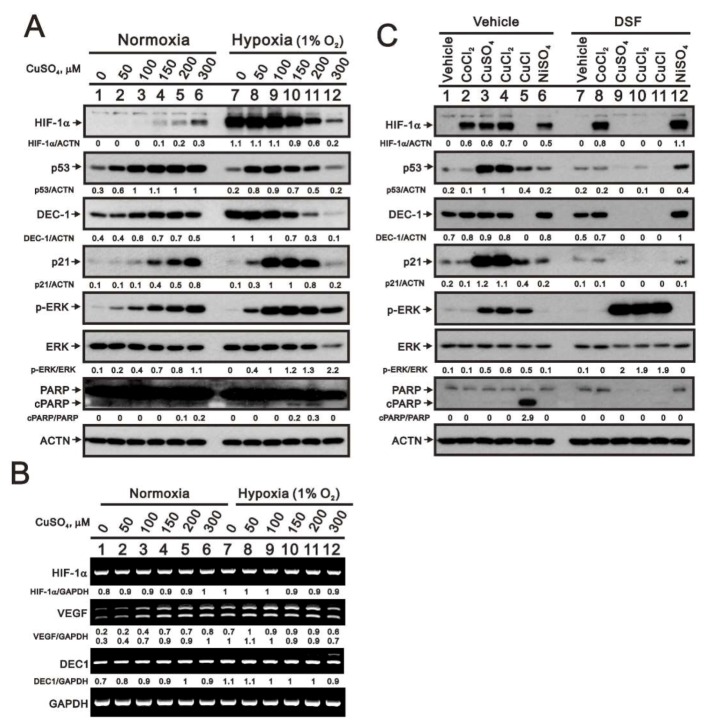
Effects of copper sulfate on expression of HIF-1α in normoxic and hypoxic HeLa cells. (**A**,**B**) HeLa cells were incubated for 6 h with the indicated concentrations of copper sulfate under normoxia and hypoxia (1% O_2_). (**A**) Cell lysates were subjected to western blot analysis using antibodies against HIF-1α, p53, DEC1, p21, total and phosphorylated (p)-ERK, and total and cPARP. (**B**) RT-PCR analysis for *HIF-1α*, *VEGF*, and *DEC1*. ACTN was the protein loading control; *GAPDH* mRNA was the mRNA loading control. (**C**) HeLa cells were incubated for 8 h with the indicated compounds (300 μM), after which cell lysates were subjected to western blot analysis using antibodies against HIF-1α, p53, DEC1, p21, total and p-ERK, and total and cleaved PARP. ACTN was the protein loading control. The results are representative of three independent experiments. Protein and PCR bands were quantified through pixel density scanning and evaluated using ImageJ, version 1.44a (http://imagej.nih.gov/ij/). The fold (shown above the bands) was normalized to the internal control protein (ACTN) or gene (*GAPDH*). Phosphorylated ERK after normalization to total ERK protein and cPARP fragment after normalization to full-length PARP protein are shown as fold changes.

VEGF, which is regulated in part by HIF-1α, stimulates angiogenesis within tumors under hypoxic conditions [15]. Given that the copper chelator tetrathiomolybdate is an anti-angiogenic agent currently in clinical trials [25,26,27], we examined the induction of HIF-1α by copper sulfate in HeLa cells and compared its effect with that of cobalt chloride, a well-known hypoxia mimetic agent [28,29]. Under normoxic conditions, higher concentrations of copper sulfate increase levels of HIF-1α protein, but not its mRNA (Figure 5A,B), which suggests copper sulfate may inhibit HIF-1α protein degradation. Under hypoxic conditions, however, higher concentrations of copper sulfate reduced levels of HIF-1α protein, though not its mRNA. We also observed that copper-induced increases in HIF-1α protein levels under normoxia were associated with increases in *VEGF* mRNA expression, while the copper-induced reduction in HIF-1α protein levels under hypoxia were associated with lower *VEGF* mRNA levels. Similarly, the levels of DEC1 (differentiated embryonic chondrocyte gene 1) and ERK protein were slightly increased by copper sulfate under normoxia and suppressed under hypoxia. By contrast, *DEC1* mRNA levels were unaffected by copper sulfate or hypoxia (Figure 5B). We also observed the induction of p53 and p21 proteins and increases in the ratio of phosphorylated (p)-ERK to total ERK by copper sulfate in normoxic HeLa cells. Under hypoxia, however, the levels of these proteins were increased at lower copper concentrations but suppressed at higher concentrations (Figure 5A). A strong apoptotic signal, cleaved PARP (cPARP) fragment, was not observed.

To examine the induction of HIF-1α protein by copper sulfate in more detail, we exposed HeLa cells to each of three copper-containing compounds (copper sulfate, copper (II) chloride, or copper (I) chloride) or two hypoxia mimetic agents (cobalt chloride or nickel sulfate) (Figure 5C). Of those, only copper (I) failed to increase HIF-1α protein levels. The three copper-containing compounds also increased p53, p21, and p-ERK protein levels. Copper (I) chloride suppressed levels of DEC1 but increased the levels of cPARP fragment. The copper chelator disulfiram (DSF) selectively suppressed the effects of copper-containing compounds on levels of HIF-1α, p53, p21, DEC1, and the cPARP fragment, but had no effect on the actions of cobalt chloride or nickel sulfate. The effects of DSF highlight the specificity and functional roles of copper in HeLa cells. Notably, DSF enhanced the increase in p-ERK levels induced by copper compounds.

### 2.5. Mechanism of Copper Sulfate-Induced Protein Expression in HeLa Cells

**Figure 6:**
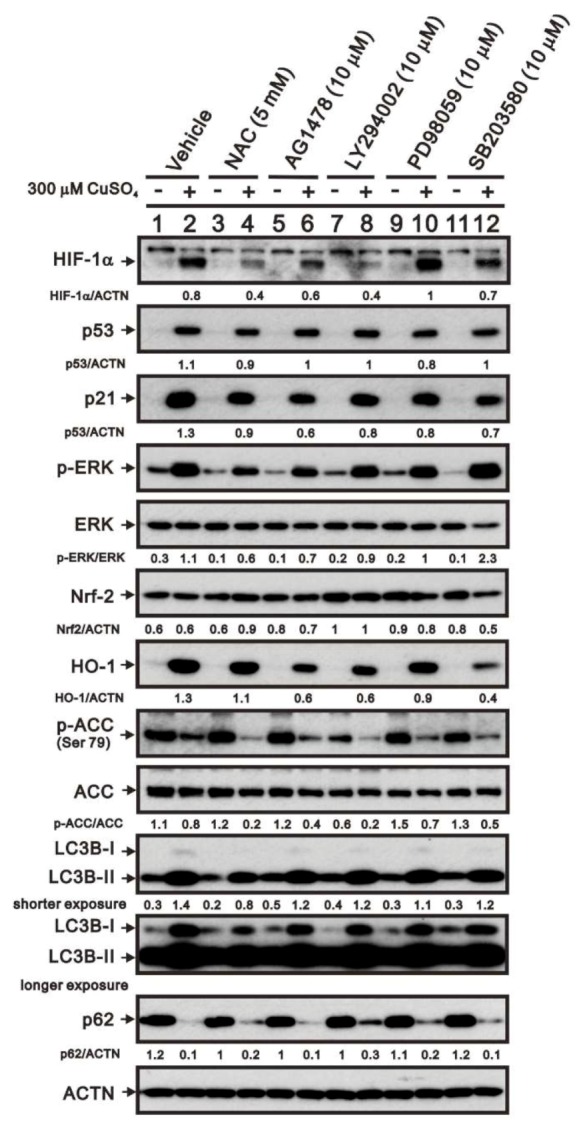
Mechanism by which copper sulfate increases HIF-1α expression. HeLa cells were incubated for 8 h with the indicated compounds in the absence (−) or presence (+) of 300 μM copper sulfate. Cell lysates were then subjected to western blot analysis using antibodies against HIF-1α, p53, p21, total and p-ERK, Nrf2, HO-1, total and p-ACC, LC3B, and p62. ACTN served as the protein loading control. The results are representative of three independent experiments. Protein bands were quantified through pixel density scanning and evaluated using ImageJ, version 1.44a (http://imagej.nih.gov/ij/). The fold (shown above the bands) was normalized to the internal control (ACTN). Phosphorylated ERK and ACC after normalization of their respective total proteins are shown as fold changes.

To determine the mechanism by which copper enhances HIF-1α protein expression, we examined the effects on copper-treated cells of the ROS scavenger NAC and various kinase inhibitors, including the tyrosine kinase inhibitor (or EGFR) AG1478, the PI3K/AKT inhibitor LY294002, the MAPK kinase inhibitor PD98059, and the p38 MAPK inhibitor SB203580 (Figure 6). Copper-induced HIF-1α expression was suppressed by NAC, AG1478, LY294002, and SB203580. Copper-induced p53 and p21 expression was unaffected by these inhibitors, though enhancement of the p-ERK/ERK ratio was suppressed by NAC, AG1478, and LY294002. The suppressive effects of NAC suggest that cytosolic ROS are involved in the copper-induced expression of these proteins. The examination of the Nrf2-HO-1 pathway showed that copper dramatically increases levels of HO-1 via a Nrf2-independent pathway. Under conditions in which cellular energy levels were depleted, AMP-activated protein kinase (AMPK) phosphorylates acetyl-CoA carboxylase (ACC) on serine-79, which leads to inhibition of its enzymatic activity for synthesis of long-chain fatty acids. We also found that copper sulfate suppresses the p-ACC/ACC ratio and that this suppressive effect was enhanced by NAC, AG1478, LY294002, PD98059, and SB203580. Microtubule-associated protein 1 light chain 3B (LC3B) and p62 are two well-established autophagy markers. During autophagy, a cytosolic form of LC3 (LC3B-I) is converted to a LC3-phosphatidylethanolamine conjugate (LC3B-II), which then associates with autophagosomal membranes. Western blotting showed that copper increased LC3B II protein levels and reduced p62 levels, suggesting that copper induces autophagy in HeLa cells. However, NAC had no consistent effect on these two proteins.

**Figure 7:**
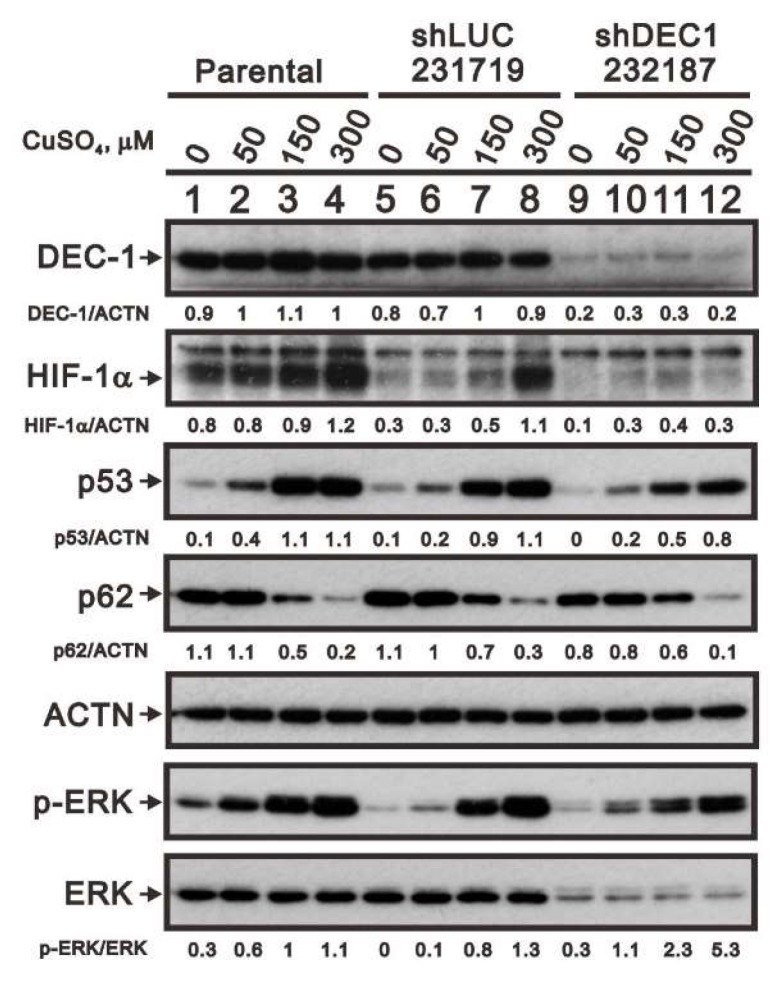
Copper sulfate acts via DEC1 to increase expression of related proteins. After *DEC1* knockdown in HeLa cells, the cells were incubated for 6 h with the indicated concentrations of copper sulfate. Cell lysates were then subjected to western blotting with antibodies against DEC1, HIF-1α, p53, total and p-ERK, and p62. ACTN served as the loading control. The results are representative of three independent experiments. Protein bands were quantified through pixel density scanning and evaluated using ImageJ, version 1.44a (http://imagej.nih.gov/ij/). The fold (shown above the bands) was normalized to internal control (ACTN). Phosphorylated ERK after normalization to total ERK protein is shown as the fold change.

We previously showed that *DEC1* is a p53 target gene involved in cellular apoptosis [30]. Here, we used *shDEC1* to knock down *DEC1* expression to assess its involvement in copper-induced HIF-1α expression (Figure 7). We found that *DEC1* knockdown decreased copper-induced upregulation of HIF-1α levels. We also found that DEC1 is associated with copper-induced increases in p53, p62, and phosphorylated ERK protein levels. Likewise, DEC1 was associated with total ERK protein expression. Consequently, following *DEC1* knockdown the p-ERK/ERK ratio was higher than control.

### 2.6. The Cell Type-Dependent Effects of Copper Sulfate on the Stability of HIF-1α

**Figure 8:**
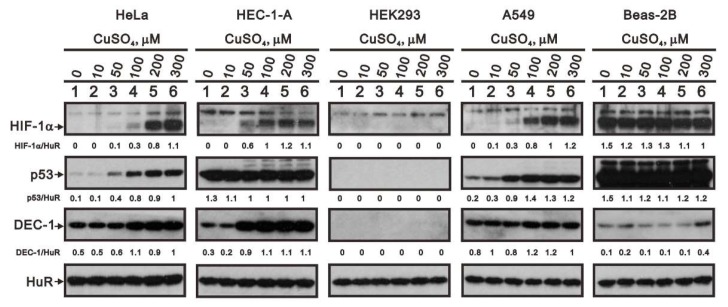
Effects of copper sulfate are cell-type specific. HeLa, HEC-1A, HEK293, A549, and Beas-2 cells were incubated for 8 h with the indicated concentrations of copper sulfate. Cell lysates were then subjected to western blot analysis using antibodies against HIF-1α, p53, and DEC1. HuR served as the protein loading control. The results are representative of three independent experiments. Protein bands were quantified through pixel density scanning and evaluated using ImageJ, version 1.44a (http://imagej.nih.gov/ij/). The fold (shown above the bands) was normalized to the internal control (HuR).

Finally, to determine whether the effects of copper sulfate were cell type-specific, we compared its effects on HIF-1α, p53, and DEC1 expression in HEC-1-A human endometrial adenocarcinoma, HEK293 human embryonic kidney, A549 human pulmonary adenocarcinoma, and Beas-2B immortalized normal human bronchial epithelial cells with those in HeLa cells (Figure 8). Our findings showed that copper selectively increased HIF-1α levels in HeLa, HEC-1-A, and A549 cells; selectively increased p53 levels in HeLa and A549 cells; and selectively increased DEC1 levels in HeLa and HEC-1-A cells. Copper had virtually no effect on the levels of these proteins in the two normal cell lines, HEK293 and Beas-2B cells. Only at 300 μM did copper slightly increase DEC1 levels in Beas-2B cells.

## 3. Discussion

**Figure 9:**
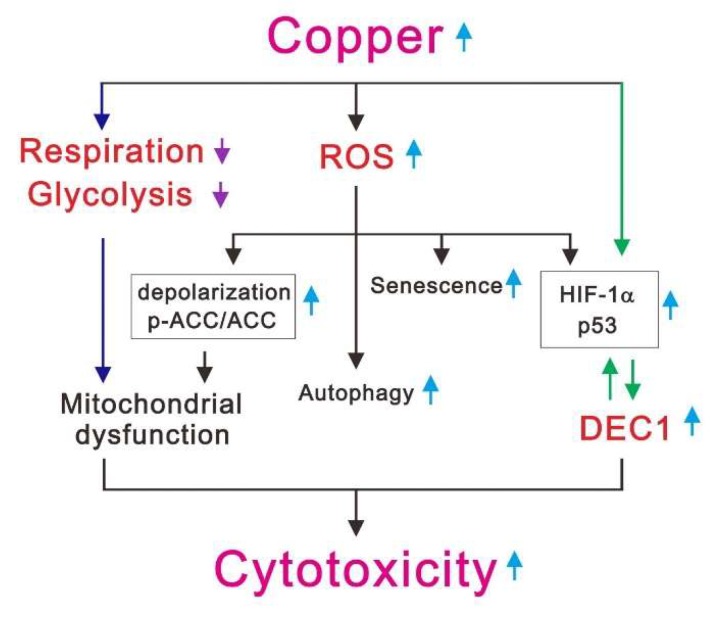
The mechanisms of copper-induced cytotoxicity in cancer cells. The cytotoxicity of copper sulfate was manifested by three parts. First, it could decrease respiration and glycolysis in cancer cells, resulting in mitochondrial dysfunction (dark blue arrows). Second, it could increase ROS levels, leading to mitochondrial dysfunction, autophagy, senescence, and upregulation of HIF-1α and p53 protein levels. Finally, it could act via DEC1 to increase expression of related proteins, such as HIF-1α and p53 (green arrows).

The levels of copper and oxidative stress are higher in cancer patients than healthy subjects [6,21,26]. ROS production via copper-mediated oxidation results in cell death [1,9,10,16]. In the present study, we first assessed the cytotoxicity of copper sulfate, which was manifested by increases in cellular apoptosis, senescence, ROS levels, mitochondrial dysfunction, autophagy, and expression of several stress proteins, including ATF3, c-Fos, DEC1, p21, p53, and HIF-1α (Figure 9). We verified the functional role of copper-induced ROS generation, using the ROS scavenger NAC, and verified the specificity of copper’s effects using the copper chelator DSF. The selective expression of HIF-1α, p53, and p21, as well as the increase in the p-ERK/ERK ratio, elicited by copper in HeLa cells was mediated by DEC1. In addition, copper’s cytotoxicity was also observed in two other cancer cell lines (HEC-1-A and A549 cells), but not in two normal epithelial cell lines (HEK293 and Beas-2B cells).

The differential effects of copper (I) and copper (II) on the levels of HIF-1α, p53, p21, and cPARP fragment suggest copper (I) induces apoptosis, which could be suppressed by the copper chelator DSF. The induction of HIF-1α, p53, and p21 expression by copper (II) was also suppressed by DSF, but there was no conversion of cPARP fragment. The ability for DSF to eliminate the effects of both copper (I) and copper (II) suggests that their actions may not be interconvertible under the conditions used in the present experiments. It is significant that we verified the involvement of the Fenton reaction using Fenton reaction inhibitors and confirmed the true copper (I or II) status using copper (I or II) probes in living cells. The efficacy of copper-binding agents against cancers remains to be determined. In that regard, in addition to inhibiting aldehyde dehydrogenase and other copper-containing enzymes, DSF decreases intracellular copper and enhances the influx of therapeutic drugs. Studies have also shown the DSF-Cu (exogenous copper II) complex is a more effective antineoplastic adjuvant than DSF alone [31,32,33,34,35]. Our present findings indicate that both copper (I) and copper (II) form a complex with DSF and that DSF further increases the abundance of p-ERK already enhanced by copper. Whether DFS/copper (I) and DSF/copper (II) act via the same signaling pathway(s) remains to be determined.

Reduced expression of antioxidant enzymes, such as SODs may contribute to the higher ROS levels seen in cancer cells [16,17]. The stabilization of HIF-1α expression under normoxia, which results from a deficiency of von Hippel-Lindau tumor suppressor protein (pVHL), and hypoxia are both reported to significantly reduce SOD2 enzymatic activity by suppressing its expression [36,37]. We found that under normoxia, higher concentrations of copper (II), but not copper (I), stabilize HIF-1α to a similar degree as cobalt chloride, a well-known hypoxia mimetic agent. The interplay between ROS and HIF-1α (or hypoxia) was evidenced by the effects of NAC, which suppressed the levels of both ROS and HIF-1α in HeLa cells. However, copper exerted an inhibitory effect on HIF-1α expression under hypoxia, suggesting that copper plays different functional roles in normoxia and hypoxia. The selective effect of copper (II), but not copper (I), on HIF-1α levels reveal that the stabilization of HIF-1α may be mediated through the inhibition of dioxygenases or related enzymes capable of hydroxylating HIF-α or other, as yet unidentified, proteins. However, details of the inhibitory effects of copper (II) on dioxygenases remain to be investigated in the future.

Our measurements of OCR, ECAR, and mitochondrial membrane potential in HeLa cells suggest copper (II) disrupts both mitochondrial respiration and glycolysis, which suggests copper (II) functions within mitochondria. However, the way that copper (II) damages mitochondria, leading to their dysfunction, remains unclear. With copper (II), there was an apparent absence of apoptosis, such as that seen with 100 μM copper (I), even when the subG1 population reached 10% in the presence of 500 μM copper sulfate. Thus, the next step will be to address the key difference between the cellular effects of copper (I) and copper (II) and how it impacts their cytotoxicity.

*DEC1* is induced by the CLOCK:BMAL1 heterodimer via the CACGTG E-box in its promoter, and DEC1 suppresses its own expression by competing with CLOCK:BMAL1 for DNA binding [38,39]. Several studies have shown that *DEC1* is regulated by pVHL, hypoxia, the UBC9/ubiquitin proteasome degradation pathway, and metformin [30,40,41,42], and that the actions of DEC1 are often mediated by direct binding to E-box elements or by protein-protein interactions with transcription factors, such as HIF-1α [43,44,45,46,47,48]. In the present study, the expression of DEC1 and HIF-1α was induced by both copper and hypoxia, which is consistent with an earlier study showing that DEC1 and HIF-1α are tumor hypoxia markers during the process of lung adenocarcinoma progression [49]. Copper (I) failed to induce HIF-1α expression and suppressed DEC1 expression. In addition, while DSF binding to copper (I) or copper (II) suppressed the expression of both HIF-1α and DEC1, knocking down *DEC1* also suppressed copper-induced HIF-1α expression. This suggests that DEC1 is involved in the copper-mediated stabilization of HIF-1α under normoxia. In addition, one study suggests that the DSF/copper complex decreases copper-induced levels of most of proteins, including HIF-1α, p53, p21, and cleaved PARP, by acting as an inhibitor of 20S proteasome activity [35].

## 4. Methods and Materials

### 4.1. Cell Culture and Reagents

HeLa and HEK293 (human embryonic kidney) cells were cultivated in Dulbecco’s modified Eagle’s medium (DMEM). A549 (human pulmonary adenocarcinoma) and Beas-2B (human bronchial epithelial) cells were cultivated in Roswell Park Memorial Institute (RPMI) 1640 medium. HEC-1-A (human endometrial adenocarcinoma) cells were cultivated in McCoy’s 5A medium supplemented with 10% fetal bovine serum (FBS) and 1% penicillin-streptomycin (Thermo Fisher Scientific, Waltham, MA, USA). *N*-acetyl cysteine (NAC), cobalt (II) chloride, copper (II) chloride, 2′,7-dichlorofluorescein diacetate (DCFH-DA), MG132, propidium iodide (PI), nickel sulfate, and thiazolyl blue tetrazlium bromide (MTT) were obtained from Sigma Aldrich (St. Louis, MO, USA). Copper (I) chloride was from Alfa Aesar (Ward Hill, MA, USA). Copper sulfate was from Serva (Heidelberg, Germany). LY294002, PD98059, and SB203580 were from Millipore (Burlington, MA, USA).

### 4.2. Cell Survival Analysis

Cells were seeded into 24-well culture plates and incubated for 1 day, after which they were exposed to different concentrations of copper sulfate in fresh DMEM for the indicated periods of time. After adding MTT solution (0.5 mg/mL in phosphate buffered saline (PBS)) to each well, the cells were incubated for 1 h at 37 °C. Dimethyl sulfoxide (DMSO; 200 μL) was then added, and the absorbance at 570 nm and 650 nm were measured using an ELISA plate reader (Multiskan EX, Thermo Fisher Scientific, Waltham, MA, USA). The control group containing cells cultured in medium only was defined as 100% cell survival.

### 4.3. Fluorescence-Activated Cell Sorting (FACS), Cell Cycle Profiles, ROS, Senescence, and Mitochondrial Membrane Potential Analyses

Cell cycle profiles were evaluated based on cellular DNA content using FACS. Cells were fixed in 70% ice-cold ethanol and stored at −30 °C overnight, after which they were washed twice with ice-cold PBS supplemented with 1% FBS and stained with PI solution (5 μg/mL PI in PBS, 0.5% Triton x-100, and 0.5 μg/mL RNase A) for 30 min at 37 °C in the dark.

The fluorescent marker DCFH-DA was used to determine intracellular ROS levels. Cells were incubated for 16 h with different concentrations of copper sulfate. Living cells were then stained with DCFH-DA (10 μM) for 40 min at 37 °C and harvested. After being washed once with PBS, the cells were evaluated using a FACSCalibur flow cytometer and Cell Quest Pro software (BD Biosciences, Franklin Lakes, NJ, USA).

For flow cytometric senescence assays, SA-β-Gal activity was measured using the fluorescent substrate 5-dodecanoylaminofluorescein di-β-d-galactopyranoside (C_12_FDG) (Invitrogen, Carlsbad, CA, USA) according to the manufacturer’s instructions. Briefly, the cells were seeded in 6-well culture plates and treated for 16 h with the indicated compounds (various concentrations of copper sulfate combined NAC or Mito-TEMPO). After the incubation, the cells were harvested, washed twice with PBS, and stained with 33 μM C_12_FDG for 15–20 min at room temperature. Fluorescence intensity was then evaluated using a FACSCalibur flow cytometer and Cell Quest Pro software (BD Biosciences, Franklin Lakes, NJ, USA).

Mitochondrial membrane potential was measured using a BD™ MitoScreen Flow Cytometry Mitochondrial Membrane Potential Detection Kit (BD Biosciences, Franklin Lakes, NJ, USA) according to the manufacturer’s instructions. In brief, HeLa cells were incubated with the different concentrations of copper sulfate and cultured to a density of less than 1 × 10^6^ cells/mL, after which they stained with JC-1 (5,5′,6,6′-tetrachloro-1,1′,3,3′-tetraethylbenzimi-dazolyl carbo-cyanine iodide) for 15 min at 37 °C in a CO_2_ incubator. The cells were then washed and JC-1 fluorescence was measured in a flow cytometer with excitation at 488 nm and emission at 530 nm (FL1-H channel) for the monomer and 580 nm (FL2-H channel) for aggregates.

### 4.4. Western Blotting

HeLa, HEC-1-A, HEK293, A549, and Beas-2B cells were lysed in RIPA buffer (100 mM Tris-HCl (pH 8.0), 150 mM NaCl, 0.1% SDS, and 1% Triton 100) at 4 °C. Proteins in the resultant lysates were separated by SDS-PAGE and analyzed by immunoblotting with antibodies against α-actinin (ACTN), ATF3, c-Fos, FASN HuR, Nrf2, p21, p53, p62 (Santa Cruz Biotechnology, Santa Cruz, CA, USA), acetyl-CoA carboxylase (ACC), p-ACC (phosphorylation at Ser 79), ERK, p-ERK, HIF-1α, LC3B, cleaved poly-ADP-ribose polymerase (cPARP) (Cell Signaling, Danvers, MA, USA), Cyclin D1 (Abcam, Cambridge, UK), HO-1 (Enzo Life Sciences, Farmingdale, NY, USA), and DEC1 (Bethyl Laboratories, Montgomery, TX, USA).

### 4.5. Reverse Transcription-Polymerase Chain Reaction (RT-PCR)

Total RNA was obtained using TRIzol (Thermo Fisher Scientific, Waltham, MA, USA) reagent according to the manufacturer’s protocol, after which 1 μg of total RNA was reverse transcribed using Murine Leukemia viruse reverse transcriptase (Epicentre Biotechnologies, Madison, WI, USA) for 60 min at 37 °C. PCR reactions were run in a GeneAmp PCR system 9700 (Thermo Fisher Scientific, Waltham, MA, USA). The PCR primers used are listed in Table 1.

### 4.6. Detection of the Oxygen Consumption Rate (OCR) and Extracellular Acidification Rate (ECAR)

The cellular OCR and ECAR were measured using a Seahorse XF24 bioenergetic assay according to the manufacturer’s instructions (Agilent, Santa Clara, CA, USA). The procedural details have been described previously [30]. Briefly, HeLa cells were suspended in DMEM containing 10% FBS, seeded onto an XF24 microplate, and incubated for 3 days. XF24 bioenergetic assays were initiated by removing the exhausted medium and replacing it with sodium bicarbonate-free DMEM (pH 7.4) containing 2% FBS and 2% horse serum (HS). The OCR and ECAR were detected at a steady state. Oligomycin (1 μM), carbonyl cyanide 4-[trifluoromethoxy] phenylhydrazone (FCCP; 0.5 μM), and rotenone/antimycin (0.5 μM) were then injected sequentially into wells to determine the maximal and non-mitochondrial respiration rates.

### 4.7. DEC-1 mRNA Interference

Lentiviral vectors, containing *DEC1* and *LUC*-shRNA, were purchased from the National RNAi Core Facility (Academia Sinica, Taipei, Taiwan). HeLa cells were infected with the indicated retroviruses or lentiviruses in selection medium, containing 2 μg/mL polybrene. The procedural details have been described previously [30,50]. The silencing efficacy was verified in Western blot assays.

### 4.8. Statistical Analysis

The values are expressed as the mean ± SD of at least three independent experiments. All comparisons between groups were made using Student’s *t*-tests. Statistical significance was set at *p* < 0.05.

## 5. Conclusions

In this study, we focused on the ROS stress induced by copper sulfate in cancer cells. Our findings suggest that copper, not only induces ROS production, it also mediates cellular senescence and mitochondrial dysfunction; increases expression of stress-related transcription factors, including p53, ATF3, c-Fos, HIF-1α, and DEC1; and increases autophagy. In addition to ROS generation and mitochondrial dysfunction, our work provides novel insight into the potential role of copper-containing compounds in antitumor applications.

## Figures and Tables

**Table 1:** PCR primers used in this study.

Gene Name	Primer Sequence (5′→3′)
***cyclin D1***	Forward: 5′-ATGGAACACCAGCTCC-3′ Reverse: 5′-TCAGATGTCCACGTCCCGC-3′
***DEC1***	Forward: 5′-GTACCCTGCCCACATGTACC-3′ Reverse: 5′-GCTTGGCCAGATACTGAAGC-3′
***GAPDH***	Forward: 5′-CTTCATTGACCTCAACTAC-3′ Reverse: 5′-GCCATCCACAGTCTTCTG-3′
***p21***	Forward: 5′-CTGAGCCGCGACTGTGATGCG-3′ Reverse: 5′-GGTCTGCCGCCGTTTTCGACC-3′
***p53***	Forward: 5′-CTCTGACTGTACCACCATCCACTA-3′ Reverse: 5′-GAGTTCCAAGGCCTCATTCAGCTC-3′
***ATF3***	Forward: 5′-ATGGGTGCCCCGACGTTG-3′ Reverse: 5′-AGAGGCCTCAATCCATGG-3′
***c-Fos***	Forward: 5′-GACTACGAGGCGTCATCCTC-3′ Reverse: 5′-GCTCTGGTCTGCGATGGGGCC-3′
***VEGF***	Forward: 5′-GGACATCTTCCAGGAGTACC-3′ Reverse: 5′-GTTCCCGAAACCCTGAGGG-3′

## Abbreviations

ACC	AMP-activated protein kinase
ACTN	α-actinin
AMPK	AMP-activated protein kinase
C_12_FDG	5-dodecanoylaminofluorescein di-β-D-galactopyranoside
DCFH-DA	2′,7-dichlorofluorescein diacetate
DEC1	Differentiated embryonic chondrocyte gene 1
DMEM	Dulbecco’s modified Eagle’s medium
DSF	Disulfiram
FACS	Fluorescence-activated cell sorting
FASN	Fatty acid synthase
FBS	Fetal bovine serum
FCCP	Carbonyl cyanide 4-(trifluoromethoxy) phenylhydrazone
H_2_O_2_	Hydrogen peroxide
HIF-1α	Hypoxia-inducible factor 1 alpha
HO-1	Heme oxygenase 1
JC-1	5,5′,6,6′-tetrachloro-1,1′,3,3′-tetraethylbenzimi-dazolyl carbo-cyanine iodide
LC3B	Microtubule-associated protein 1 light chain 3B
MTT	Thiazolyl blue tetrazlium bromide
NAC	*N*-acetyl cysteine
OCR	Oxygen consumption rate
cPARP	Cleaved Poly-ADP-ribose polymerase
PBS	Phosphate buffered saline
pVHL	Von Hippel-Lindau tumor suppressor protein
PI	Propidium iodide
ROS	Reactive oxygen species
RPMI	Roswell Park Memorial Institute
RT-PCR	Reverse transcription-polymerase chain reaction
SOD	Superoxide dismutase
VEGF	Vascular endothelial growth factor
